# A Toolbox for Functional Analysis and the Systematic Identification of Diagnostic and Prognostic Gene Expression Signatures Combining Meta-Analysis and Machine Learning

**DOI:** 10.3390/cancers11101606

**Published:** 2019-10-21

**Authors:** Johannes Vey, Lorenz A. Kapsner, Maximilian Fuchs, Philipp Unberath, Giulia Veronesi, Meik Kunz

**Affiliations:** 1 Functional Genomics and Systems Biology Group, Department of Bioinformatics, University of Würzburg, 97074 Würzburg, Germany; johannes.vey@uni-wuerzburg.de (J.V.); maximilian.fuchs@uni-wuerzburg.de (M.F.); 2 Institute of Medical Biometry and Informatics, University of Heidelberg, Im Neuenheimer Feld 130.3, 69120 Heidelberg, Germany; 3 Center of Medical Information and Communication Technology, Erlangen University Hospital, 91054 Erlangen, Germany; lorenz.kapsner@uk-erlangen.de; 4 Chair of Medical Informatics, Friedrich-Alexander University of Erlangen-Nürnberg, 91058 Erlangen, Germany; philipp.unberath@fau.de; 5 Unit of Thoracic Surgery, Humanitas Research Hospital, Via Manzoni 56, 20089 Rozzano (Milan), Italy; giuliaveronesi1@gmail.com

**Keywords:** Bioinformatics tool, R package, machine learning, meta-analysis, biomarker signature, gene expression analysis, survival analysis, functional analysis

## Abstract

The identification of biomarker signatures is important for cancer diagnosis and prognosis. However, the detection of clinical reliable signatures is influenced by limited data availability, which may restrict statistical power. Moreover, methods for integration of large sample cohorts and signature identification are limited. We present a step-by-step computational protocol for functional gene expression analysis and the identification of diagnostic and prognostic signatures by combining meta-analysis with machine learning and survival analysis. The novelty of the toolbox lies in its all-in-one functionality, generic design, and modularity. It is exemplified for lung cancer, including a comprehensive evaluation using different validation strategies. However, the protocol is not restricted to specific disease types and can therefore be used by a broad community. The accompanying R package vignette runs in ~1 h and describes the workflow in detail for use by researchers with limited bioinformatics training.

## 1. Introduction

The combination of biomarkers (so-called biomarker signature) allows us to represent the information contained in biological samples and fluids, supporting clinical decisions [1]. Numerous studies demonstrated the clinical usefulness of diagnostic (disease detection) and prognostic (disease outcome) gene-expression signatures derived from microarray analysis [2,3]. For instance, MammaPrint is a 70 gene-expression prognostic signature for powerful disease outcome prediction in breast cancer [4]. The diagnostic miR-Test shows promising results for lung cancer early detection [5].

However, reliable clinical signatures are restricted by dataset availability, which often reduces their statistical power [3,6]. Artificially increasing the number of samples by combining different large cohorts using dataset merging (meta-analysis) is a beneficial solution enabling numerous insights into biological systems [7,8,9,10], but methods for biomarker signature identification are currently limited. For instance, the R packages virtualArray [11] and inSilicoMerging [12] allow virtual array merging but are no longer available and are removed from current Bioconductor releases [13]. On the other hand, database tools such as SurvExpress [14] and SurvMicro [3] allow for the assessment of a prognostic signature in cancer. Similarly, the miRpower tool provides survival analysis for miRNA biomarkers using expression data from 2178 breast cancer patients [15] and GOBO based on 1881 breast cancer dataset [16], whereas the Kaplan-Meier Plotter enables outcome analysis for ovarian cancer based on 1287 patients [17]. However, these tools focus on specific diseases and signature types. More importantly, they allow only online analysis, requiring a gene list as input, but not the calculation of signatures from in-house data. These characteristics limit them as stand-alone tools, suggesting new bioinformatics approaches.

Machine learning (ML) approaches have been demonstrated to be useful in medicine. For example, studies report that ML could be used in cancer diagnosis [18] and prognosis [19] as well as prediction of optimal cancer therapies [20]. It can also improve the prediction of heart failure readmissions [21]. 

Regularized Generalized Linear Models such as L1/L2 regularized and Elastic net regression address overfitting and aim to balance between accuracy and simplicity of a model [22,23]. The Least Absolute Shrinkage and Selection Operator (LASSO) uses L1 regularization, whereas Elastic net implements a mixture of L1 and L2 regularization. Applying these regularization techniques to fit a Generalized Linear Model is widely used for feature selection and is extremely effective when dealing with high dimensional data, which contains a large set of features. The LASSO model allows the shrinkage of the coefficients of the less contributive variables to be exactly zero (the penalty term L1-norm) [22]. Thereby, the tuning parameter lambda controls the strength of the penalization (regularization). The cross-validation calculates the lambda.min value, which reflects the model with the lowest prediction error, whereas the lambda.1se value represents a simpler model but within one standard error of the optimal model. However, the LASSO regression tends to over-regularization and has limited strength in highly correlated data. 

The Elastic net balances between LASSO (L1-norm) and ridge penalties (L2-norm) shrinking some coefficients close to zero (like ridge) and some exactly to zero (similar to LASSO) [23]. This model is powerful in datasets with e.g., correlations between variables. For this, the hyper-parameter alpha controls the mixing between the two penalty techniques (alpha = 0 for ridge; alpha = 1 for LASSO) and can be set manually between 0 and 1 to receive a model with the desired size, whereas the parameter lambda fine-tunes the amount of shrinkage [23]. Therefore, the Elastic net is a powerful method for feature selection and can operate with continuous as well as categorical features.

Several statistical methods have been developed for survival data analysis [24,25]. The Cox Proportional Hazard model is the most popular multivariate approach to investigate survival time in medical research [24,26]. It describes the relation between event incidence (hazard function, survival probability) and covariates [24,25]. 

We previously introduced a sample merging approach that is compatible with current Bioconductor releases [27]. It allows the use of datasets from databases such as Gene Expression Omnibus (GEO), The Cancer Genome Atlas (TCGA), and own experimental data [27], greatly enhancing the number of available datasets for analysis. Starting from this, we developed a protocol for the systematical calculation of diagnostic and prognostic gene signatures that combines (i) meta-analysis (multiple dataset integration) with (ii) functional gene expression analysis and (iii) ML approaches. Our aim was to develop a general framework for functional analysis and signature calculation with high predictive performance that is not restricted to specific disease types and can therefore be used by a broad community. 

## 2. Results

### 2.1. Meta-Analysis (Dataset Download, Normalization, Merging, Batch Effect Correction)

We demonstrate the workflow of our toolbox by analyzing three lung cancer datasets from microarray profiling downloaded from the GEO database. The datasets GSE18842 (45 non-tumor, 46 tumor samples) and GSE19804 (60 tumor/60 non-tumor samples) were downloaded (getGEO) and are already GCRMA normalized deposited in GEO. For the datasets GSE19188 (91 tumor/ 65 non-tumor samples), we downloaded the raw data (CEL files). The files were imported into the R environment and subsequently GCRMA normalized (resulting “ExpressionSet” object) using the gcrma package version 2.56.0 [28] (Figure S1; datasets from Chip GPL570, Affymetrix Human Genome U133 Plus 2.0). The merged dataset contained 54,675 transcripts and 367 samples (197 tumor/170 non-tumor samples; no gene transcripts were excluded during the merging procedure). The batch effect detection using a gPCA (Top) and the resulting boxplot of the merged dataset after batch effect correction (Bottom) are shown in Figure S2.

### 2.2. Functional Gene Expression Analysis

**Figure 1:**
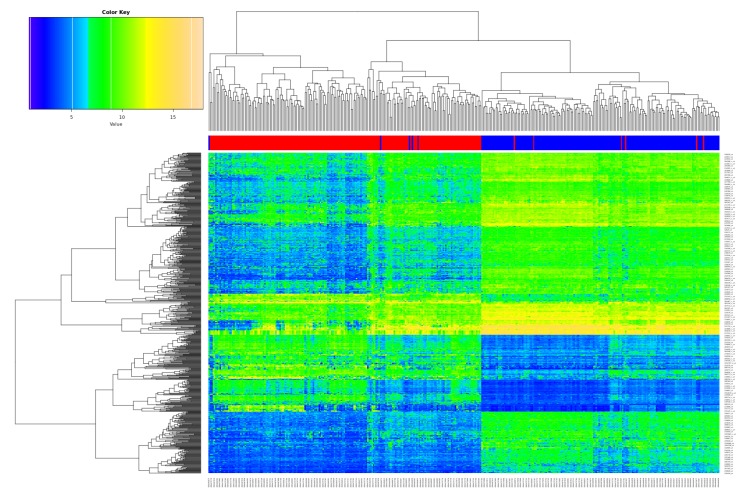
Overview of the differentially expressed genes (DEGs). Heatmap of the 699 DEGs derived from the meta-analysis with the merged datasets GSE18842, GSE19804 and GSE19188 (samples on the x-axis, DEGs on the y-axis; red color represents tumor, blue non-tumor (control) samples).

The differentially expressed genes (DEG) analysis after batch correction resulted in 699 significantly deregulated transcripts (Table S1; *q*-value < 0.05, logFC > 2/< −2 as standard criterion for selecting significantly deregulated genes [29]). Figure 1 shows the heatmap of the DEGs, illustrating a clear separation of tumor and non-tumor samples in two expression clusters. Many of them are known key players in lung cancer, for instance G Protein-Coupled Receptor Kinase 5 (GRK5) [30], Solute Carrier Family 46 Member 2 (SLC46A2) [31], and Collagen Type XI Alpha 1 Chain (COL11A1) [32] function as oncogenic factors in lung cancer.

**Figure 2:**
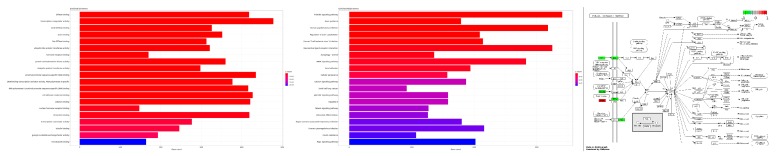
Functional Gene Ontology (GO) term and pathway enrichment analysis. (**Left**) Enriched GO terms including adjusted p-value as color code. (**Middle**) Enriched Kyoto Encyclopedia of Genes and Genomes (KEGG) pathways including adjusted p-value as color code. (**Right**) The phosphatidylinositol 3-kinase (PI3K)-Akt signaling pathway (hsa04151) from the KEGG database including the differentially expressed genes (DEGs) are highlighted considering differential expression.

We further tested the 699 DEGs for enriched Gene Ontology (GO) terms and Kyoto Encyclopedia of Genes and Genomes (KEGG) pathways (Figure 2, enriched GO terms and KEGG pathways after False Discovery Rate (FDR) control are shown). For instance, the analysis shows enriched functions such as hormone receptor binding and protein serine/threonine kinase activity (Left) and enriched pathways such as Phosphatidylinositol 3-Kinase-Akt (PI3K-Akt) signaling pathway and Mitogen-Activated Protein Kinase (MAPK) signaling pathway (Middle). Moreover, specific pathways depending on the interest of the users can be further investigated. As an example, we show the PI3K-Akt signaling pathway (hsa04151) from the KEGG database including the expression values of the involved DEGs (Figure 2, Right; red: upregulated, green: downregulated).

### 2.3. Calculation of Diagnostic and Prognostic Signatures

**Figure 3:**
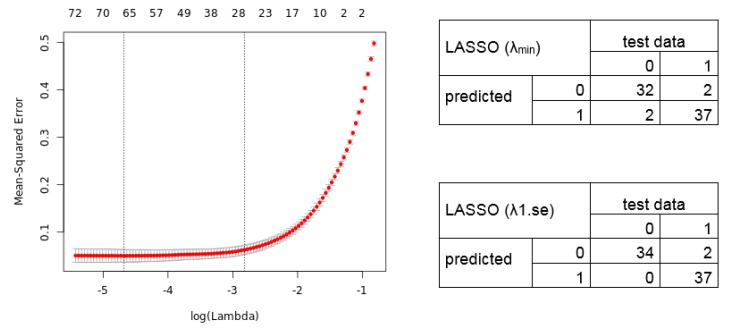
Mean-Squared error for 10-fold cross-validation according to the log of lambda on the training lung cancer dataset. (**Left**) The cross-validation errors and the upper and lower standard deviation along the lambda values of the Least Absolute Shrinkage and Selection Operator (LASSO) regression model are shown. The vertical dotted lines represent the two selected lambdas. The lambda.min value (left line) minimizes the prediction error (MSE), whereas lambda.1se (right line) gives the most regularized model (most simple model within one standard deviation of the optimal model). Values above the plot show the number of variables included in the model. (**Right**) Confusion matrix depicting the diagnostic potential of the signatures validated on the test dataset (0 = healthy, 1 = tumor).

We next analyzed the merged dataset (54,675 transcripts) for a diagnostic signature. We divided the merged dataset into a training dataset (80%; 294 samples) and test dataset (20%, 73 samples). We used a L1/L2 regularized logistic regression to fit a Generalized Linear Model in order to perform a feature selection to include only the potentially most predictive variables (here genes) in the model. The 10-fold cross-validation results in a lambda of 0.009260 and 0.059521 (Figure 3; alpha = 1). The lambda.min identifies a selection of 64 transcript variables (55 unique gene symbols) whose coefficients were not forced to be zero, whereas the lambda.1se identifies a 26 gene transcript signature (24 unique gene symbols) (Table S2). Figure 3 shows the cross-validation error (Left) and the confusion matrix (Right) for the calculated LASSO signatures predicting the test data samples. 

**Figure 4:**
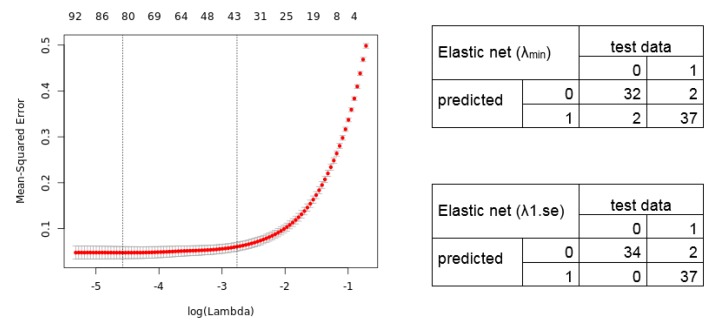
Elastic net regression model. (**Left**) The plot displays the 10-fold cross-validation errors and the upper and lower standard deviation along to the lambda values of the Elastic net regression model. The vertical dotted lines represent the two selected lambdas. The lambda.min value (left line) minimizes the prediction error (MSE), whereas lambda.1se (right line) gives the most regularized model (most simple model within one standard deviation of the optimal model). Values above the plot show the number of variables included in the model. (**Right**) Confusion matrix depicting the diagnostic potential of the signatures validated on the test dataset (0 = healthy, 1 = tumor).

We further applied the Elastic net regression. The 10-fold cross-validation shows a lambda of 0.010288 and 0.063129 (alpha = 0.9). Notably, we manually set alpha = 0.9 as the grid search for lambda (0 to 0.0001 with 100 intervals) calculates an alpha = 0.1 (lambda = 0.521401), resulting in a signature without an improved predictive performance. The Elastic net regression model identified, for lambda.min, an 80 gene transcript signature (69 unique gene symbols), and for lambda.1se, a 41 transcript signature (36 unique gene symbols) (Table S2). The calculated cross-validation error (Left) and resulting confusion matrix (Right) of the predicted test data samples by the Elastic net model are shown in Figure 4. 

**Figure 5:**
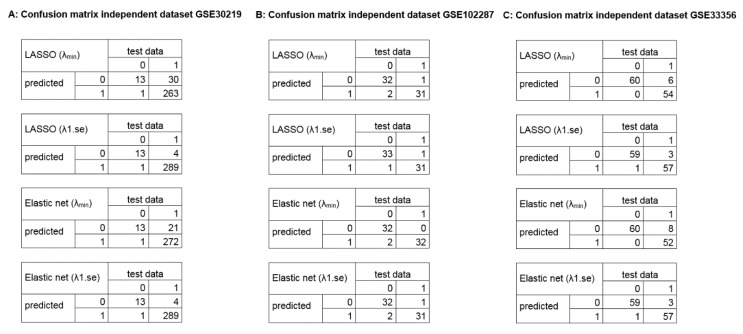
Confusion matrices of the identified diagnostic signatures in independent datasets. The plots illustrate the diagnostic classification using the identified signatures in the independent validation dataset (54,675 genes; 0 = healthy, 1 = tumor). (**A**) GSE30219, 293 lung cancer samples, 14 non lung cancer samples. (**B**) GSE102287, 32 lung cancer samples, 34 non lung cancer samples. (**C**) GSE33356, 60 lung cancer samples, 60 non lung cancer samples.

To address overfitting and reduce model instability, the framework allows to include further datasets for validation. We validated the gene signatures in three independent datasets (GSE30219, 293 lung/14 non lung cancer samples; GSE102287, 32 lung/34 non lung cancer samples; GSE33356, 60 lung/60 non lung cancer samples; 54,675 genes). The GSE30219 contains <5% non-cancerous samples, whereas the GSE102287 and GSE33356 are more balanced validation datasets. The results of the validation are depicted in Figure 5 (confusion matrices) and Supplementary Table S3 (diagnostic values), showing a high diagnostic power to classify between lung cancer and non-lung cancer samples.

After determining the diagnostic signature, we tested for a relevant prognostic signature. For this, we analyzed the significant influence of the 699 DEGs on the patient survival outcome using a Univariate Cox Proportional Hazard Model (82 patient survival outcome data from GSE19188). The Cox regression analysis revealed 22 DEGs that have a significant influence (effect size) on the patient survival (Table S4; *p*-value < 0.05). We found known lung cancer drivers such as Lipoprotein Lipase (LPL) [33] and CC Chemokine Receptor 2 (CCL2) [34]. 

Next, we trained the prognostic 22 gene classifier using an algorithm comparing the expression profiles between tumor and healthy samples of the merged datasets GSE18842 and GSE19804 (we excluded GSE19188 for classification to avoid selection bias, as it is the dataset for the identification of survival correlated genes). We additionally validated the identified 22 prognostic gene signature in two independent datasets (GSE30219: 278 from 293 patients with survival data, GSE50081: 181 patients with survival data) to evaluate its impact on the patient outcome. Here, we tested whether the 22 gene signature can classify patients with high and low mortality risk. Therefore, we classified the patient samples into high risk and low risk groups using the trained classifier. 

**Figure 6:**
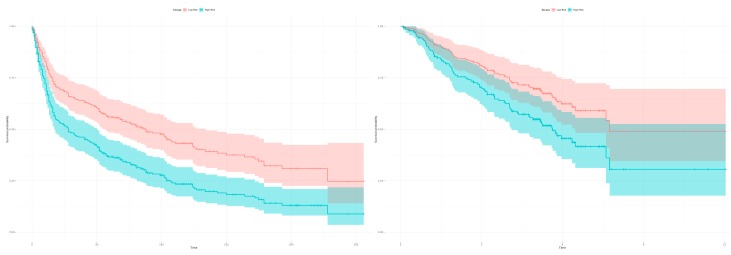
Kaplan-Meier estimators with computed 95% confidence interval to evaluate the patient classification in high and low risk groups deploying the 22 gene signature on two independent datasets. The classification in high and low risk groups is based on the expression profiles between tumor and healthy samples of the merged datasets (GSE18842, GSE19804). (Left) The plot shows a classification in high and low risk groups for the 293 patients from the validation dataset GSE30219 based on the 22 survival correlated genes (*p*-value = 0.0002166; low risk: 121 samples, high risk: 172 samples, number of events/deaths: 188). (Right) The 22 gene signature can classify the 181 patients in the validation dataset GSE50081 in high and low risk groups (*p*-value = 0.02919; low risk: 88 samples, high risk: 93 samples, number of events/deaths: 75).

The Kaplan-Meier estimators in Figure 6 demonstrate the significant patient classification achieved regarding high and low risk groups for the 22 genes in the validation dataset GSE30219 (Left: *p*-value = 0.0002166) and GSE50081 (Right: *p*-value = 0.02919). This indicates that the identified 22 gene classifier reflects a common prognostic signature of dominant tumor factors that can differentiate between high and low risk tumor disease.

## 3. Discussion

Our intention was to develop a general and easy to use toolbox that identifies reliable diagnostic and prognostic signatures including the important steps of data augmentation and validation, especially for users with limited bioinformatics resources. It is therefore a step-by-step protocol rather than an improved algorithm or ML method approach.

The tool applies a comparison between the two ML models LASSO and Elastic net, which aim to balance between accuracy and simplicity of a model. LASSO and Elastic net regularization are well-established methods for gene expression analysis, allowing to construct predictive models from datasets with non-linear and large dimensional variable numbers [21]. Especially for generalization of data with additive variable and outcome dimensions or a low number of training datasets they generate predictive results similar to complex ML algorithms [19]. Complex ML approaches such as support vector machines, neural networks, random forest, and gradient boosting algorithms allow unbiased predictive models using complex variable selection and huge datasets but tend to overfitting in the identification of large biomarker combinations [1,19,35]. However, the combinations of biomarkers show better discriminatory power for clinical decision support rather than a single biomarker [1]. 

The use of ML implies the need for a substantial amount of data in order to train the model, in which the integration of different datasets might be required. However, gene expression analysis often suffers from selection bias, poor sample quality, and poor sample size estimation, influencing the statistical power and validity of downstream analysis [1,36,37]. Combing different gene expression datasets using meta-analysis has been shown to increase statistical power and overcome selection biases including the identification of diagnostic and prognostic biomarkers [7,8,9,10,38,39,40]. However, differentially gene expression selection using meta-analysis is mostly based on univariate p-value statistics which introduces the problem to identify sets of genes with non-redundant information and to find the correct number of genes that describe the data [8]. This limits application for diagnostic and prognostic signatures that integrate several feature selections and covariates such as patient characteristics (e.g., survival) and histology [8]. We overcome this by implementing a meta-analysis for the integration of multiple gene expression datasets into a merging array and then applied ML methods to identify biomarker signatures from datasets with non-linear and large dimensional variable numbers.

Several studies calculate signatures using ML approaches, but often fail during independent validation stages [36]. To overcome overfitting and reduce model instability, we identified a classifier in the training dataset and applied a comprehensive evaluation using different validation strategies—in particular, a split sample, internal validation (cross-validation) and testing in independent datasets. Moreover, we applied a multiple-testing correction using the Benjamini and Hochberg method and set a stringent q-value of 0.05. We recommend using a stringent *q*-value (can be set by the user) to reduce the false positives and find real biologically deregulated genes but also considering sample size and power estimation approaches based on statistical and clinical significance [1,41]. This strengthens the robustness for the biomarker signature identification capability and validity for clinical usefulness.

In our example, the identified gene signatures from two different ML models show a high diagnostic power and might be promising for the clinic to classify between lung cancer and non-cancer samples. The confusion matrix for the LASSO and Elastic net regression models are similar. Comparing the calculated signatures shows a common set of 12 transcripts (12 unique gene symbols), and similar accuracy and predictive performance. However, this is of course not always the case. For example, studies in breast cancer reported two independent prognostic signatures identified with similar approaches showing only few common genes, which were experimentally validated [42]. This illustrates that different mathematical models should be applied to find the most reliable signature rather than using only one method. Hence, using several methods reduces false positive results even for challenging datasets and avoids misclassification in experimental and clinical testing. This strengthens the validity and clinical usefulness of signatures extracted from large gene expression datasets.

The common gene set contains known cancer markers. For instance, TMEM106B has been shown to be a valuable marker of lung cancer metastasis [43], whereas COL10A1 [44] plays a diagnostic role of circulating extracellular matrix-related proteins. However, LGR4 [45] is known as a diagnostic marker in prostate cancer. This highlights that our analysis approach allows the identification of reliable diagnostic signatures. The next step is then to validate and iteratively refine the marker signature derived from our tool in prospective clinical studies to find an optimal biomarker signature, with the help of more complex ML models. 

The significance and novelty of the toolbox lies in its functionality as an „all-in-one tool”: it offers an analysis path combining meta-analysis with functional gene expression analysis and robust diagnostic and prognostic signature calculation. The code is implemented in an R package. The four main functions—*sigidentDEG*, *sigidentEnrichment*, *sigidentDiagnostic*, and *sigidentPrognostic*—are wrapper functions around all included smaller functions to execute the analysis steps. However, these can also be run separately, depending on the interests of the users.

The toolbox benefits from its generic design and modularity. We designed it for Affymetrix as a widely used microarray profiling platform [46] and illustrate the generality of the approach using lung cancer gene expression datasets (tumor/healthy) downloaded from the GEO database. The generic design of the tool allows the analysis of different types of gene expression signatures, e.g., mRNA, lncRNA, and miRNA. Furthermore, it supports analysis in front of the high biological complexity of tumors, for instance analysis of tumor subtypes and heterogeneity. 

We demonstrated the method’s power to be applied to datasets containing a large number of gene probes using the Affymetrix HG-U133 Plus 2.0 platform. However, the merging algorithm is not restricted to this platform, allowing the potential integration of other popular microarray profiling platforms such as HG-U133A, HG-U133B, and HG-U133A 2.0. Moreover, the modularity of the framework allows the future incorporation of additional platforms, such as Illumina, but also other high-throughput data such as genomic, proteomic, metabolomic, and radiomic data. For instance, the Elastic net model shows applicability to genome-scale data such as the identification of genomic markers of drug sensitivity [8,47]. Indeed, the implementation of this complex data requires programming skills and is therefore recommended only for experienced users. Such a broad applicability is in principle possible but was not the intention of the current version of the framework and should be the focus of future work. Further efforts should also focus on the integration of the toolbox into a web application to provide its functionality to users without R programming skills. 

Existing tools such as SurvMicro [3] and SurvExpress [14] allow for the online validation of prognostic signatures, but are restricted to datasets from TCGA and limited to cancer. Our toolbox has the advantage to be disease independent and allows the integration of data from TCGA and GEO, but also from in-house experiments. 

The framework from Hughey et al. 2015 identifies a diagnostic signature combining meta-analysis with an Elastic net regression [8]. This approach is similar to our method, but our tool calculates prognostic signatures as a further relevant biomarker signature for clinical application. Additionally, the regularization methods LASSO and Elastic net can be applied for the aim of feature selection to identify variables correlated to the desired response variable. The toolbox also integrates an automated method to identify DEGs, including a summary table with gene annotations and functional enrichment analysis. In this way, our method can also be used to perform a functional DEG analysis from merged datasets without the calculation of signatures. In conclusion, the user-friendly R package, the all-in-one functionality, and modularity make the framework useful to a broad community.

## 4. Materials and Methods

**Figure 7:**
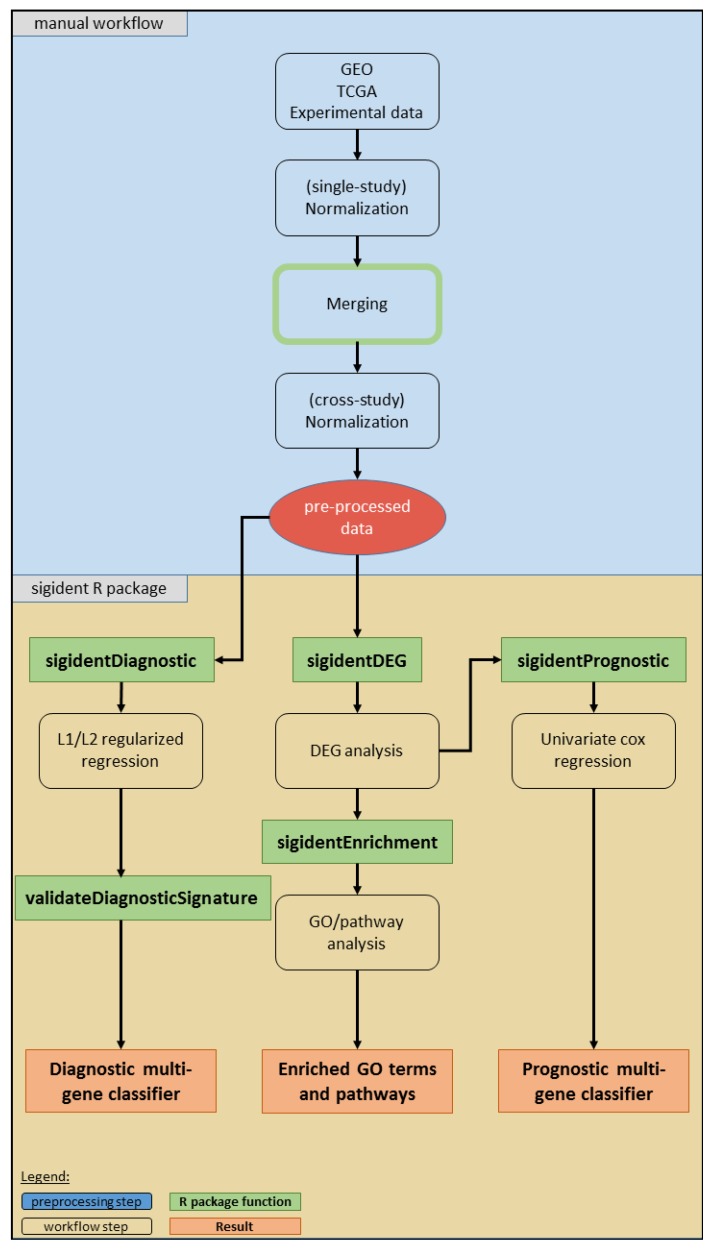
Overview of the workflow of our toolbox. The boxes show the analysis steps, colored rectangles represent the R package functions and results (see legend).

Figure 7 illustrates the workflow of our toolbox. It has been developed and tested on R version 3.6.1 (R Bioconductor version 3.9). We implemented the code into the R package “sigident” (https://gitlab.miracum.org/clearly/sigident), which provides the four main functions—*sigidentDEG*, *sigidentEnrichment*, *sigidentDiagnostic*, and *sigidentPrognostic.* The whole workflow is documented in detail in the R package vignette.

Supplementary Table S5 lists the used R packages. The newly created “sigident” R package integrates a (i) meta-analysis (multiple dataset integration), (ii) functional gene expression analysis, and (iii) the calculation of statistically robust multi-gene signature combinations. As an application example, we used lung cancer datasets from the GEO database (GSE18842, GSE19804, and GSE19188). After merging, we divided the dataset into a training (80%) and test (20%) dataset for the calculation of the diagnostic signature. Moreover, we validated the diagnostic signature in three independent datasets (GSE30219, GSE102287, GSE33356). For the prognostic signature, we performed a survival analysis using the GSE19188 which includes survival information and validated the signature in two independent datasets (GSE30219, GSE50081).

For the meta-analysis (dataset download, normalization, merging) and the functional gene expression analysis (analysis for DEGs, heatmap), we used our previously published sample merging approach, which is based on a modified code of the inSilicoMerging package combined with the limma package [27]. This approach has been developed further in order to integrate it into the “sigident” R package framework. In detail, it uses the R package GEOquery version 2.52.0 for dataset downloading [48], gcrma package version 2.56.0 for CEL file loading, background correction, quantile normalization, and log2-transformation [28], Biobase package version 2.44.0 for integration of standardized data structures [13], gplots package version 3.0.1.1 for graphical representation [49], and the limma package version 3.40.6 for the DEG analysis [50]. We extended the code by detecting batch effects using a guided principal component analysis from the gPCA package version 1.0 [51]. For batch effect correction, we used empirical Bayes framework applying the ComBat function from the sva package version 3.32.1 [52] considering different groups (tumor, ctrl). As a DEG analysis is known to generate false positive results [36], we applied a multiple-testing correction using the Benjamini and Hochberg approach to control the FDR [53]. We used a stringent q-value (adjusted FDR value) of 0.05. 

Furthermore, for the DEGs we added a functional gene ontology (GO) and KEGG pathway enrichment analysis using the goana and kegga functions from the limma package (Entrez IDs as input). A further GO and pathway over-representation test is implemented using the clusterProfiler package version 3.12.0 [54] (including FDR control, DEGs are mapped to their Entrez-IDs as input), whereas specific pathways can be further investigated using the pathview package version 1.24.0 [55]. 

The calculation of statistically robust multi-gene signature combinations focuses on diagnostic and prognostic signatures. For diagnostic signatures, we used the LASSO and Elastic net penalty as implemented in the R package glmnet version 2.0.18 [56]. The hyper-parameter alpha can manually be set to a value between 0 and 1 or can automatically be calculated in combination with the tuning parameter lambda based on cross-validation and a grid search applying the wrapper function train as implemented in the caret package version 6.0.84 [57]. In the case of a fixed value for alpha, lambda is determined by 10-fold cross-validation, and a leave-one-out cross-validation is also possible. For calculation of the Receiver Operating Characteristics (ROC) and the Area Under the Curve (AUC) value of the ML models we used the pROC package version 1.15.3 [58].

For the prognostic signature detection we applied a survival and risk assessment analysis using a Cox Proportional Hazard Model as implemented in the survival R package version 2.44.1.1 [59]. The Cox Proportional Hazard regression analysis identifies genes that have a significant effect size on the survival outcome. To generate a prognostic signature, we applied a classification algorithm that assigns patients in high and low risk groups based on the expression profiles of the identified survival correlated genes between tumor and non-tumor samples. Survival curves were plotted using the survminer package version 0.4.5 [60].

## 5. Conclusions

We developed an efficient toolbox for the identification of diagnostic and prognostic gene signatures. It is the first R package tool that combines meta-analysis with gene expression analysis and ML approaches for the systematical calculation of statistically robust gene signatures. This helps to reduce study biases and improves the statistical power for the identification of reliable signatures from large sample cohorts. Importantly, the tool is not restricted to a specific disease. We believe that our toolbox will be useful for the research community and opens new windows for an effective analysis of data and a better clinical management of diseases.

## Data Availability

The toolbox is publicly available as R package under the URL https://gitlab.miracum.org/clearly/sigident.

## Supplementary Materials

The following are available online at https://www.mdpi.com/2072-6694/11/10/1606/s1, Figure S1. Boxplots of the GCRMA normalized expression data (training and test dataset). The dataset GSE18842 contains 45 non-tumor and 46 tumor samples (Left), the GSE19804 dataset 60 non-tumor and 60 tumor samples (Middle) and the GSE19188 dataset 65 non-tumor and 91 tumor samples (Right). (GCRMA normalized; datasets from Chip GPL570, Affymetrix Human Genome U133 Plus 2.0); Figure S2. Plots for the batch effect detection using the gPCA (training and test dataset). (Top) The merged dataset contains 54,675 transcripts and 367 samples (170 non-tumor (control), 197 tumor samples; no gene transcript were excluded during the merging process). The plots show the gPCA before (Left) and after (Right) batch correction. (Bottom) Boxplots of the merged datasets before (left) and after (right) batch effect removal; Table S1. List of the 699 DEGs. The table lists the 699 DEGs (q-value < 0.05, logFC > 2/< −2) in the merged dataset after batch effect correction (517 unique gene symbols of total 699 ID transcripts); Table S2. Overview of the calculated signatures from the LASSO and Elastic net regression models; Table S3. Predictive parameters of the identified diagnostic signatures in the independent dataset. (A) GSE30219, 293 lung cancer samples, 14 non lung cancer samples. (B) GSE102287, 32 lung cancer samples, 34 non lung cancer samples. (C) GSE33356, 60 lung cancer samples, 60 non lung cancer samples. total: 54,675 genes; Table S4. List of the 22 DEGs. The table lists the 22 DEGs that are significantly associated with the survival outcome (affy gene ID according to affy_hg_u133_plus_2; p-value < 0.05; 20 unique genes of total 22 transcripts, two variants of each DLC1 and LPL; HR > 1: poor prognosis, HR < 1: good prognosis, HR = 1: no effect); Table S5. Overview of the used R packages (for details see https://gitlab.miracum.org/clearly/sigident).
