# Supplementary material for: A Toolbox for Functional Analysis and the Systematic Identification of Diagnostic and Prognostic Gene Expression Signatures Combining Meta-Analysis and Machine Learning
Source: Cancers (Basel). 2019 Oct 21;11(10):1606. doi: 10.3390/cancers11101606 (PMC6827106; doi:10.3390/cancers11101606)
Supplement: Supplementary file 1 [file cancers-11-01606-s001.zip › Suppl_rev/supplementary materilas.docx]

Supplementary Materials


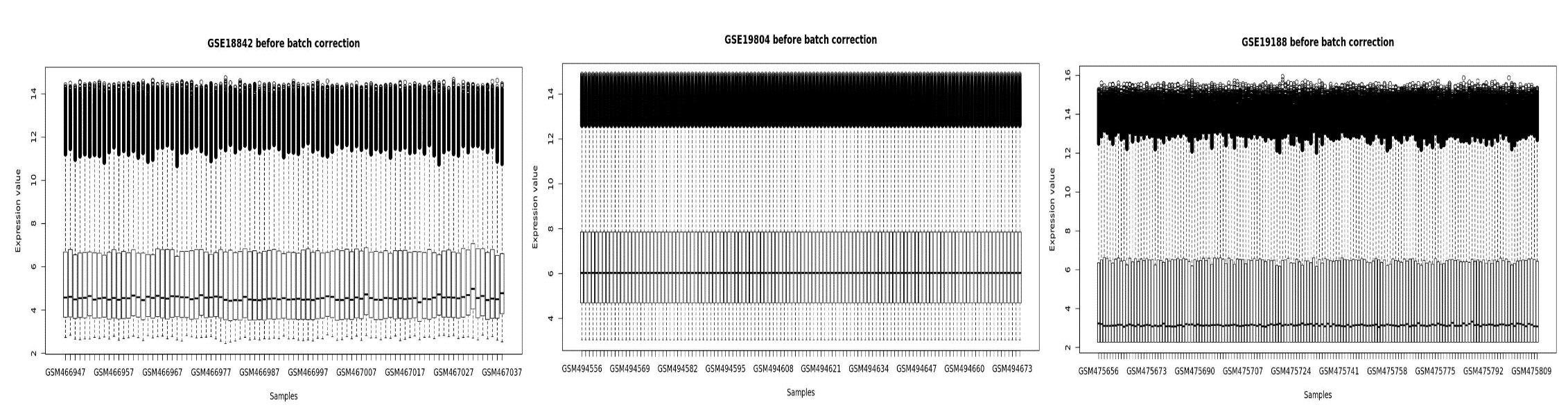


**Figure S1.** Boxplots of the GCRMA normalized expression data (training and test dataset). The dataset GSE18842 contains 45 non-tumor and 46 tumor samples (Left), the GSE19804 dataset 60 non-tumor and 60 tumor samples (Middle) and the GSE19188 dataset 65 non-tumor and 91 tumor samples (Right). (GCRMA normalized; datasets from Chip GPL570, Affymetrix Human Genome U133 Plus 2.0).


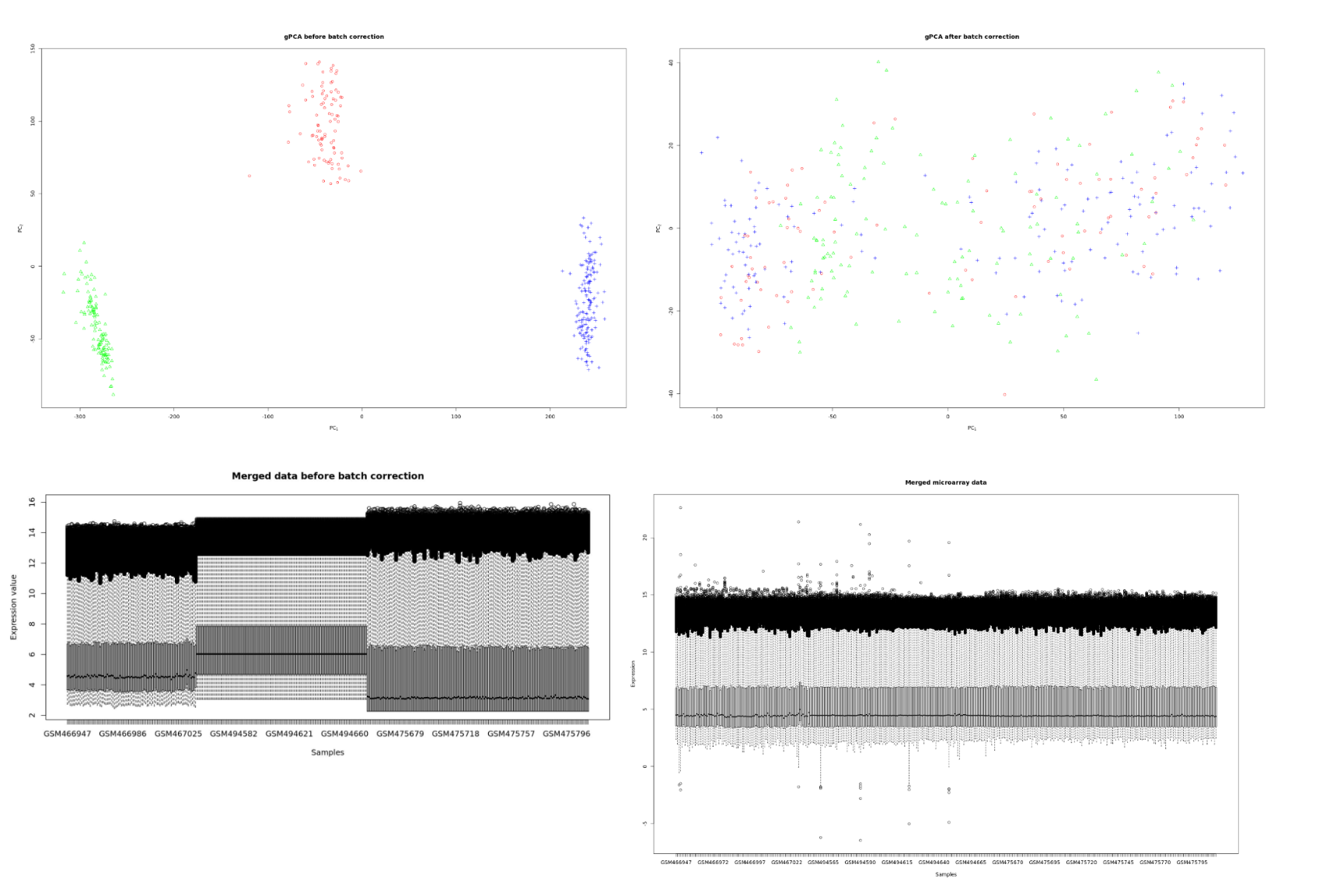


**Figure S2.** Plots for the batch effect detection using the gPCA (training and test dataset). (Top) The merged dataset contains 54,675 transcripts and 367 samples (170 non-tumor (control), 197 tumor samples; no gene transcript were excluded during the merging process). The plots show the gPCA before (Left) and after (Right) batch correction. (Bottom) Boxplots of the merged datasets before (left) and after (right) batch effect removal.
